# Mycotoxin concentrations in rice from three climatic locations in Africa as affected by grain quality, production site, and storage duration

**DOI:** 10.1002/fsn3.959

**Published:** 2019-02-11

**Authors:** Erasmus N. Tang, Sali A. Ndindeng, Jude Bigoga, Karim Traore, Drissa Silue, Koichi Futakuchi

**Affiliations:** ^1^ Faculty of Science, Department of Biochemistry University of Yaoundé‐I Yaoundé Cameroon; ^2^ Africa Rice Center Bouake Côte d’Ivoire; ^3^ Africa Rice Center Saint‐Louis Sénégal

**Keywords:** Africa, community interventions, food safety, healthy food systems, mycotoxin, rice

## Abstract

Information on the mycotoxin contamination of rice in Africa is limited although the risk of contamination is high. In this study, domestic milled rice processed by actors using suboptimal methods was purchased and total fumonisin (FUM), zearalenone, and aflatoxin concentrations determined at 0, 90, and 180 days after storage. Three different climatic locations, Cotonou (Benin) in the Guinea savanna, Yaoundé (Cameroon) in the Tropical forest, and N'diaye (Senegal) in the Sahel, were selected as storage sites. Subsets of the samples collected from Glazoue (Benin), Ndop (Cameroon), and Dagana (Senegal) were stored in plastic woven bags under room conditions in the respective sites with or without calcium oxide (burnt scallop shell—BSS, 0.1% w/w) treatment. Multivariance analysis showed that FUM concentration was positively influenced by the duration of storage only while zearalenone concentration was negatively influenced by relative humidity and head rice but positively by impurities. Zearalenone concentration was also influenced by sample collection/storage location, processing type, and duration of storage. Aflatoxin concentration was influenced negatively by storage room temperature and head rice but positively by impurities and chalky grains. In addition, aflatoxin concentration was influenced by collection/storage location and processing type. BSS treatment followed by storage for 6 months had no effect on the concentration of the three assessed mycotoxins. Strategies to reduce the risk of mycotoxin contamination in study sites will include the improvement of physical rice quality through better pre‐ and postharvest practices and proper packaging of both treated rice and untreated rice in hermetic systems before marketing and storage.

## 1 INTRODUCTION

The production and processing of rice, which is considered a strategic staple in sub‐Sahara Africa (SSA), mostly employ suboptimal methods often resulting to insufficiencies in quantity and quality (Mapiemfu et al., 2017; Ndindeng et al., 2015) of the end products. The rice value chain, comprising the pre‐ and postharvest segments, has unit operations aiming at producing premium quality milled rice that is safe for both, human and animal consumption. In each of these segments, the physicochemical, nutritional, and economic value of the milled rice can be reduced by microbial invasions especially in the humid tropics (Gummert, Balingbing, Barry, & Estevez, 2009). Farmer's practices and environmental conditions that enhance insect propagation, microbial proliferation, and mycotoxin production during these production and processing stages include the following: the use of disease‐infected seeds, the nonelimination of disease‐infected plants during production, poor harvesting, threshing, drying, parboiling, and milling methods favoring grain damage and/or their contamination (Amponsah, Addo, Dzisi, Moreira, & Ndindeng, 2017; Mapiemfu et al., 2017; Ndindeng et al., 2015) and storage of grains in systems that favor the re‐absorption of moisture or expose them to high oxygen levels (Fleurat‐Lessard, 2017; Magan, Hope, Cairns, & Aldred, 2003), which promote microbial proliferation. Enormous quality deterioration occurs during storage (Majumder, Bala, Arshad, Haque, & Hossain, 2016) including mycotoxigenic secretion by contaminants predominantly represented by the genera *Aspergillus,* *Fusarium, *and *Penicillium* (Makun, Dutton, Njobeh, Mwanza, & Kabiru, 2011; Makun, Gbodi, Akanya, Salako, & Ogbadu, 2007; Reddy, Reddy, & Muralidharan, 2014). Other important contaminants described by Makun et al. (2007) in moldy rice in Nigeria include genera of *Rhizopus, Mucor, Trichoderma, Helminthosporium, Cladosporium, Curvularia*, and *Alternaria*. When grain moisture and temperature exceed critical safe storage limits (> 14% and 25–37ᵒC respectively), field initial fungi inoculums will start to proliferate and initiate the quality deterioration process known as moldy grains (Christensen & Kaufmann, 1965; Magan, Medina, & Aldred, 2011). Mycotoxins of great risks to human and animal health are aflatoxins (B1, B2, G1, G2) and ochratoxin A secreted by *Aspergillus *spp. and *Penicillium* spp.; fumonisins (B1, B2, B3), deoxynivalenol, and zearalenone (ZEA) secreted by the *Fusarium *spp.; and patulin produced by *Penicillium* spp. (Audenaert, Vanheule, Höfte, & Haesaert, 2014; Gaag et al., 2003; Iqbal, Jinap, Pirouz, & Ahmad Faizal, 2015). Huge adverse effects on humans exposed to cereal grains with high mycotoxins content have been reported. A lethal outbreak of human aflatoxicosis following the intake of moldy maize led to 317 diagnosed cases and 125 deaths in Kenya in 2004 (Center for Disease Control & Prevention, 2004). Aflatoxins (AFLAs) especially AFLA B1, classified as human carcinogens by the International Agency for Research on Cancer (IARC, 1993), act as immune suppressors and can lead to acute illness or even death (Williams et al., 2004). They are widely reported as hepatotoxic, teratogenic, and mutagenic. Fumonisin (FUM) B1 is a potent cancer promoter, classified as possible human carcinogen (IARC, 1993) causing in vivo nephrotoxicity and hepatotoxicity (Gelderblom et al., 1988, 2002; Gelderblom, Kriek, Marasas, & Thiel, 1991). Deoxynivalenol and ZEA are not classified as carcinogenic in humans (IARC, 1993) but have been reported to exert immune suppression and estrogenic effects (Kostro et al., 2011; Meky, Hardie, Evans, & Wild, 2001). Despite efforts toward improving rice production in sub‐Saharan Africa (SSA) because of its strategic importance in diets of millions of people, controlling its fungal contamination remains challenging especially as most pre‐ and postharvest operations are rudimentary and manual. In addition, rice is mostly sold unpackaged or stored in plastic woven or jute bags, thus increasing the rate of fungal contamination and proliferation. Rice is also by itself a suitable culture medium for mycotoxigenic fungi especially when poorly stored as total AFLA, ZEA, ochratoxin A, deoxynivalenol, and citreoviridin are produced and accumulated (Almeida et al., 2012). In a recent review on worldwide occurrence of mycotoxins, the highest level of AFLAs was detected in polished rice from Africa (1,642 ppb) with an incidence of 50% compared to 850 ppb in corn from Asia with an incidence of 63% (Lee & Ryu, 2017). These authors suggested that environmental conditions prevailing in Africa enhance fungal growth and aflatoxin production. From the same report, ochratoxin A and ZEA occurrence were highest in rice from Africa (1,164 ppb and 1,169 ppb, respectively) while deoxynivalenol (112.2 ppb) in rice came third behind wheat (303 ppb) and corn (436 ppb). Most of the data were collected on stored and marketed food crops from Ivory Coast (Sangare‐Tigori et al., 2006), Nigeria (Makun et al., 2011, 2007), Tunisia (Bensassi, Zaied, Abid, Hajlaoui, & Bacha, 2010; Ghali, Hmaissia‐Khlifa, Ghorbel, Maaroufi, & Hedili, 2008; Zaied et al., 2009; Zaied, Zouaoui, Bacha, & Abid, 2012), Kenya (Muthomi, Ndung'u, Gathumbi, Mutitu, & Wagacha, 2008), Cameroon (Abia et al., 2013; Njobeh et al., 2010), Malawi (Matumba, Monjerezi, Khonga, & Lakudzala, 2011), and Morocco (Juan, Zinedine, Idrissi, & Mañes, 2008; Zinedine et al., 2006, 2007).

Mycotoxin distribution in milled rice fractions has been reported at different levels. For instance, in brown and white rice from the Philippines, highest AFLA contamination was respectively reported at 2.7 and 8.7 ppb (Sales and Yoshizawa (2005). In Sri Lanka, Bandara, Vithanege, and Bean (1991) detected levels of 185 and 963 ppb of AFLA B1 and AFLA G1 in parboiled rice. Sangare‐Tigori et al. (2006) and Makun et al. (2011) reported the occurrence of AFLAs, FUMs, ochratoxin A, and deoxynivalenol in white rice from SSA. In SSA, rice is mostly consumed in the white and parboiled milled forms but there is no comparative report on their mycotoxin contents. It is however suspected that rudimentary parboiling conditions may favor mycofloral contamination and mycotoxin accumulation. Rudimentary artisanal parboiling, involving prolonged soaking (24–48 hr.) in water at atmospheric conditions and insufficient sun drying, produces inferior quality parboiled rice with moisture content greater than the recommended 12%–14% safe level for storage (Ndindeng et al., 2015). Processed paddy samples from these technologies are therefore susceptible to toxigenic fungal colonization and mycotoxin production. When milled white and parboiled rice are poorly stored (inappropriate temperature and relative humidity conditions) for long durations, toxigenic fungal infestation, proliferation, and mycotoxin production are favored (Choi et al., 2015).

The use of resistant varieties, best practices in pre‐ and postharvest handling, physical, and chemical treatment have been reported as strategies to reduce postharvest losses by pest in stored grains and mycotoxin reduction to below safe levels (Sheahan & Barrett, 2017) especially in wheat (Cheli, Pinotti, Rossi, & Dell'Orto, 2013) and rice (Choi et al., 2015). Limited information exists on how physical grain qualities and processing type (parboiled or white milled) affect mycotoxin accumulation at different climatic locations in SSA. This study sought to investigate the effects of sample collection/storage location, relative humidity, and temperature in the storage room, physical grain qualities, processing type (white vs. parboiled), calcium oxide treatment (moisture absorber), and storage duration on the concentration of total FUM, ZEA, and AFLA in rice. As a contribution toward mycotoxin control, burnt scallop shell (BSS) powder at 0.1% was tested for their efficacy on mycotoxigenic fungal growth and mycotoxin accumulation in rice.

## 2 MATERIALS AND METHODS

### 2.1 Sample collection treatment and storage

Two processed rice types; white and parboiled milled collected in March 2014 were used for the study. The two types of milled rice produced and processed in the three study sites originated from popular farmer association processing units located in Glazoué (Benin), Ndop (Cameroon), and Dagana (Senegal). The most popular rice variety: IR‐841–5‐3, TOX‐3145‐TOC‐38–2‐3, and SAHEL‐108 (IR 13,240–108–2‐2–3) respectively in Glazoué, Ndop, and Dagana was selected. A 60 kg pooled sample of each rice type with initial moisture content of 14% was collected from rice mills in each zone, properly mixed, and stored in two replicates of 15 kg each as treated and untreated samples from April to September 2014. For treated samples, the rice was thoroughly mixed with 0.1% calcium oxide (BSS powder) as recommended by the manufacturer (Toyo SC Trading Co., Ltd, Tokyo, Japan). This product was expected to act as a desiccant to reduce the equilibrium moisture content of the samples. BSS has been approved in Japan as a food additive and safe for human consumption. Samples from Glazoue, Ndop, and Dagana were stored respectively in Cotonou (Benin) located in the Guinea savanna (Subhumid agroecological zone), Yaoundé (Cameroon) located in the Tropical forest (humid agroecological zone), and N'diaye‐Senegal located in the Sahel (semi‐arid agroecological zone) (HarvestChoice, 2009). Samples for storage were put in plastic woven bags (commonly used in the region) and placed on pallets (one replicate on the other). The storage rooms were treated with insecticide powder (0.05% antoukar super; pirimiphos‐methyl 16 g/kg, permethrin 3 g/kg: DP) to eliminate insects during storage**. **Room temperature and relative humidity were measured every hour during the storage period using a Dickson TP125 temperature/humidity data logger (Dickson, Addison, IL). Table 1 a and b illustrates the set up and handling of treated and untreated samples up to the point of mycotoxin quantification.

**Table 1:** (a) Setup of treated samples for the evaluation of mycotoxins in milled rice

Type of samples	Actions taken at		
	Day 0	Day 90	Day 180
Treated samples (Point 0 measurement)	1. Collected samples from popular processing units; 2. Treated samples with BSS 3. Ground samples and stored in the refrigerator at 4°C	1. Samples kept at 4°C	1. Remove samples from the refrigerator same day as those of 180 days; 2. Determine mycotoxins
Treated samples (3‐month measurement)	1. Collected samples from popular processing units; 2. Treated samples with BSS 3. Put in plastic woven bags and stored at ambient conditions	1. Ground samples and store them at 4°C	1. Remove samples from the refrigerator same day as those of 180 days; 2. Determine mycotoxins
Treated samples (6‐month measurement)	1. Collected samples from popular processing units; 2. Treated samples with BSS 3. Put samples in plastic woven bags and stored at ambient conditions	1. Continued to keep samples at ambient conditions	1. Ground samples and store at 4° 2. Remove samples from the refrigerator and determine mycotoxins

Type of samples	Actions taken at		
	Day 0	Day 90	Day 180
Untreated samples (Point 0 measurement)	1. Collected samples from popular processing units; 2. Ground samples and stored in the refrigerator at 4°C	1. Samples kept at 4°C	1. Remove samples from the refrigerator same day as those of 180 days; 2. Determine mycotoxins
Untreated samples (3‐month measurement)	1. Collected samples from popular processing units; 2. Put in plastic woven bags and stored at ambient conditions	1. Ground samples and store them at 4°C	1. Remove samples from the refrigerator same day as those of 180 days; 2. Determine mycotoxins
Untreated samples (6‐month measurement)	1. Collected samples from popular processing units; 2. Put samples in plastic woven bags and stored at ambient conditions	1. Continued to keep samples at ambient conditions	1. Ground samples and store at 4° 2. Remove samples from the refrigerator and determine mycotoxins

### 2.2 Physical grain quality characterization of rice samples

The moisture content of samples at the time of collection was measured with a PQ‐510 Single Kernel Moisture Meter (Kett Electric Laboratory, Tokyo, Japan) and expressed as a percentage. Impurities expressed as a percentage were evaluated by manually separating and weighing foreign matter in a 200 g sample. The percentage of whole grains (head rice) was determined by separating broken from whole grain in a 100 g sample using a Test Rice Grader (Satake, Hiroshima, Japan). Grain chalkiness, a measure of the granular packing of starch within the rice endosperm, was measured using the S21 Rice Statistical Analyzer (LKL Tecnologia, Brazil) as previously described by Ndindeng et al. (2015).

### 2.3 Isolation and identification of fungi

For fungal isolation, samples (200 g) were randomly collected from several locations in each bag from top to bottom using a grain collector after 0, 3, and 6 months. For the identification of mycoflora from rice grains, 10 g of 10 randomly selected subsamples of rice grains from each type was surface‐disinfected using 10% sodium hypochlorite solution for 2 min, rinsed thrice with sterile distilled water, and then dried under a laminar flow hood on sterilized Whataman paper. For each sample, 15 whole grains were plated in triplicates (45 grains) on 90 mm in diameter Petri dishes containing Nutrient Broth Yeast Extract Agar or NBY (8 g nutrient broth, 2 g yeast extract, 0.5 g KH_2_PO_4_, 2 g K_2_HPO_4_, 2 g glucose, 15 g agar powder, and 1,000 ml distilled sterile water). After incubation for seven days at 25°C (12‐hr fluorescent light and 12‐hr darkness), the resulting fungal colonies were individually subcultured onto potato dextrose agar and diagnosed by mounting conidia and mycelium on glass slides in water, examined under a light microscope, and identified using the keys of Barnett and Hunter (1972). Individual grains showing different fungi were isolated and recorded. Isolates of *Fusarium* spp. were identified using guidelines of McClenny (2005) and Nelson, Toussoun, and Marasas (1983) while *Aspergillus* spp. and *Penicillium* spp. followed the description of Pitt and Hocking (1997).

### 2.4 Isolation and identification of bacteria

Enumeration of bacterial strains was as described by Dossou and Silue (2018). It included subculturing and purification of single colonies on Peptone Sucrose Agar (PSA) medium (10 g peptone, 10 g sucrose, 1 g glutamic acid, 15 g Agar powder, and 1,000 ml sterile distilled water), DNA extraction for Multiplex PCR to diagnose *Xanthomonas oyrzae* pathovars (Lang et al., 2010), PCR for the identification of *Pantoea spp*. (Kini, Agnimonhan, Afolabi, Milan et al., 2017; Kini, Agnimonhan, Afolabi, Soglonou, Silué et al., 2017), *Sphingomonas* (Kini, Agnimonhan, Dossa, Soglonou et al., 2017), and Bacillus species.

### 2.5 Sampling, extraction and quantification of mycotoxins

A 200 g pooled sample collected from the top, middle, and bottom of the bag of each of the treatments was ground to fine powder in a grinder (UDY cyclone mill; Fort Collins, Co., USA) fitted with a fine sieve of 0.5‐mm mesh size (Zohoun, Tang et al., 2018). The extraction and quantification of AFLA, FUM, and ZEA from rice flour were done using the AFLA, FUM, and ZEA competitive enzyme‐linked immunosorbent assay (ELISA) plate kit methods (Beacon Analytical Systems, Inc. Saco, ME). Briefly, ZEA was extracted by vigorously shaking 20 g of rice flour in 100 ml of 70% methanol (i.e.7:3 v/v, methanol: water) solution for 3 min. The slurry was allowed to settle for another 3 min and the supernatant filtered through a Whatman GF/A filter paper (GE Healthcare, USA) into a 50‐ml sterile beaker. Duplicate of 1 ml of the filtrate was diluted with 4 ml of 70% methanol and used for the ELISA assay. The diluted solutions and all the reagents were brought to room temperature prior to the assay. For the ZEA quantification, the diluted filtrates from the samples (100 µl) together with equal volumes of ZEA standards corresponding to 0, 0.02, 0.05, 0.25, and 1.0 µg/ml (ppm) were pipetted into the mixing wells using clean pipette tips. Thereafter, 200 µl of zearalenone‐horseradish pyroxidase (ZEA‐HRP) conjugate was dispensed into the wells and content gently homogenized by pipetting the solution in and out with a multichannel pipettor and then 100 µl of the homogenate transferred into the test wells and incubated for 10 min at room temperature. The test wells were then decanted and washed 5 times by overflowing the wells with distilled water. The last wash water was removed from the wells by inverting on an absorbent paper. The HRP substrate (100 ul) was then added into the wells and incubated at room temperature for 5 min and 100 µl stop solution (1 N HCl acid) added into each well. The intensity of the resulting yellow color was spectrophotometrically measured and the absorbances read at 450 nm using a Stat fax 303 plus plate reader (Awareness Technology, Inc. Palm City, FL, USA). FUM was extracted by filling a 250‐ml conical flask containing 100 ml deionized water with 20 g of ground sample. The lid was tightly closed and vigorously mixed for 3 min and then allowed to settle for another 3 min. The supernatant was filtered through a Whatman GF/A filter paper (GE Healthcare, USA) into a 50‐ml clean beaker. The filtrate for ELISA was diluted 1:4 ml with deionized water. For quantification, equal volumes of the FUM‐HRP conjugate enzyme, sample filtrate (50 µl), and standard solutions corresponding to FUM (0, 0.3, 1, 3.0, and 6.0 µg/ml) were respectively dispensed into each test well. Thereafter rabbit anti‐FUM antibody solution was added into the corresponding test wells, and the mixture was incubated for 10 min at room temperature. The wells were washed and dried by gently tapping on absorbent paper; then, 100 µl of HRP substrate was pipetted into each well and incubated for 5 min at room temperature. The reaction was stopped by adding the stop solution (100 ul, 1 N HCl) and the intensity of the resulting yellow color was measured at 540 nm using a Stat fax 303 plus plate reader (Awareness Technology, Inc. Palm City, FL). For AFLA extraction, 50 g of the finely ground rice was mixed with 5 g of NaCl and blended for 1 min at high speed in 100 ml of 80% methanol. The supernatant was filtered through a Whatman GF/A filter (GE Healthcare, USA) and 5 ml of the filtrate was diluted with 20 ml distilled water and further filtered through a glass fiber. For quantification, equal volumes of AFLA‐HRP conjugate enzyme, sample filtrate (50 µl), and standard solutions corresponding to AFLA (0, 2.0, 7.5, 25, and 100 µg/L (ppb) were respectively dispensed into each test well. Thereafter, rabbit anti‐AFLA antibody solution was added into the corresponding test wells and the mixture was incubated for 10 min. The wells were washed and dried with an absorbent paper; then, 100 µl of the HRP substrate was added and the well incubated for 5 min at room temperature. The stop solution (100ul, 1 N HCl) was added, and the intensity of the resulting yellow color was measured at 540 nm using a Stat fax 303 plus plate reader (Awareness Technology, Inc. Palm City, FL).

### 2.6 Data analysis

Data from Dickson TP125 temperature/humidity data logger (Dickson, Addison, IL) were downloaded using the Dickson software (Dickson, Addison, IL) and imported into Microsoft Excel 365 where plots of temperature and relative humidity against time were produced. Mean equilibrium moisture content of stored rice was determined from the mean temperature and relative humidity during the storage period based on the International Rice Research Institute (IRRI) table for the determination of the equilibrium moisture content of rice during storage (http://www.knowledgebank.irri.org/postproductioncourse/index.php/storage/equilibrium-moisture-content). Data on physical grain quality characteristics of studied samples were displayed using bar charts. Box‐plots of microbial load (colonies/plate) against microbes identified in the different rice processing types were plotted. Using the absorbances of the standard samples, the log‐linear regression model in XLSTAT software for Windows (Version 18.6, 2017) (Addinsoft SARL, Paris) was used to develop equations predicting the concentration of mycotoxins from recorded absorbances of the samples (Equations (1), (2), (3) below).

(1) $$\text{FUM}\;\text{predicted}\;\text{conecntration}\;\left(\text{ppm}\right)=\exp^{(4.66-3.24*\text{Absorbance)}}$$

(2) $$\text{ZEA}\;\text{predicted}\;\text{concentration}\;\left(\text{ppm}\right)=\exp^{(9.17-6.26*\text{Absorbance)}}$$

(3) $$\text{AFLA}\;\text{predicted}\;\text{concentration}\;\left(\text{ppb}\right)=\exp^{\left(6.3-4.20*\text{Absorbance}\right)}$$

Multivariance analysis models were used to study the effects of quantitative variables (relative humidity and temperature in the storage room and physical grain qualities (head rice, impurities, and chalkiness) and qualitative variable (site of sample collection/storage, processing type, BSS treatment, and duration of storage) on mycotoxin concentration. For qualitative variables, Dagana/N'diaye, white milled rice, treated and stored for 6 months were used respectively as references for collection/storage location, processing type, BSS treatment, and duration of storage in the estimation of model parameters. All analyses were carried out at 5% significance level.

## 3 RESULTS AND DISCUSSIONS

### 3.1 Temperature, relative humidity in the storage room, and equilibrium moisture content

**Figure 1:**
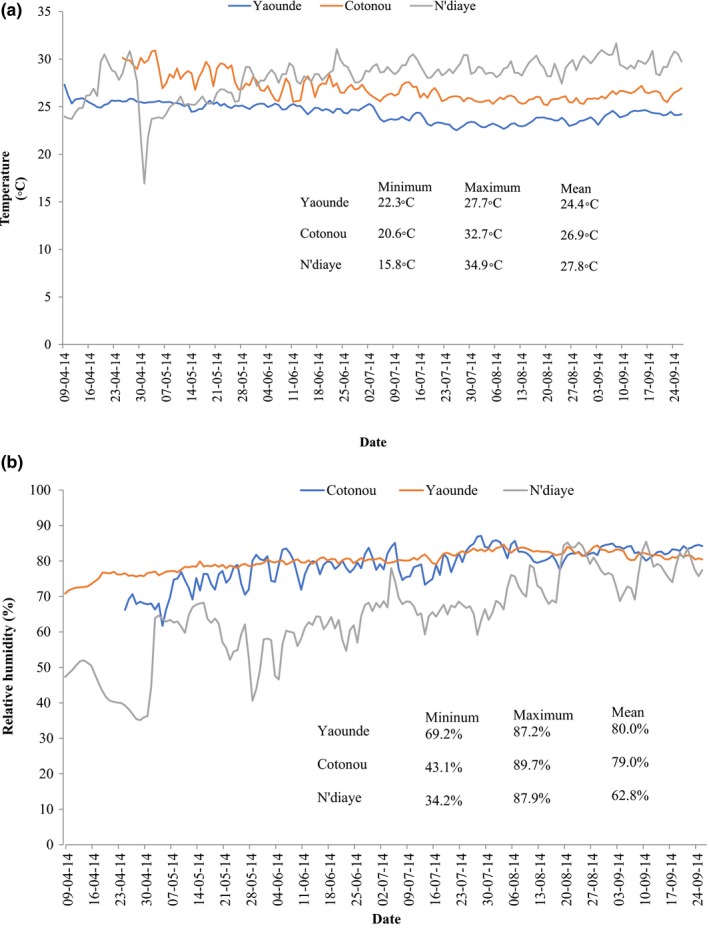
(a) Temperature and (b) Relative humidity at sites where milled rice samples were stored and later evaluated for mycotoxin contamination

Figure 1a depicts the time series of environmental temperatures over the 6 months duration in the three storage locations. Temperature and relative humidity of the storage rooms were measured with a data logger placed outside the storage bags. A minimum temperature of 19.57°C and a maximum of 31.77°C with an overall mean of 26.37°C were recorded across all locations. The minimum (15.8°C), maximum (34.9°C), and highest mean temperature (27.8°C) together with multiple fluctuations in temperature were recorded in N'diaye—Senegal. Slight fluctuations were observed in Cotonou—Benin compared to Yaoundé—Cameroon and temperatures in those two sites followed a steadier trend compared with N'diaye. The average mean temperature in Yaoundé and Cotonou was 24.4 and 26.9°C, respectively.

Figure 1b presents the time series relative humidity over the storage period in all three locations. The lowest mean relative humidity (62.8%) was recorded in the storage rooms in N'diaye, followed by Cotonou (79.0%) and the highest was in Yaoundé (80.0%). The highest fluctuations in relative humidity were recorded in N'diaye (34.2%–62.8%). In the case of Cotonou, slight fluctuations were observed between April and June compared to Yaoundé where a steady trend was observed.

In Cotonou, the mean temperature, relative humidity, and estimated equilibrium moisture content of the grains during the storage period were 79%, 26°C, and 14.7%, respectively. These values indicate that the storage of grains in woven plastic bags or other systems that allowed moisture reabsorption was unsafe. In N'diaye, the mean temperature, relative humidity, and estimated equilibrium moisture content of the grain during the storage period were 62.8%, 27.8°C, and 12.4%, respectively, indicating that the storage of grains in that location during the study period in woven plastic bags or other systems that allow moisture reabsorption was safe. In Yaoundé, the mean temperature, relative humidity, and estimated equilibrium moisture content of the grain during the storage period were 80%, 24.4°C, and 15.7%, respectively. These values indicate that the storage of grains in that location during the period of study in woven plastic bags or other systems that allow moisture reabsorption was unsafe.

Temperature and relative humidity showed an increasing trend in all storage rooms from the start to the end of storage period. Although the optimum conditions for fungal spore germination and proliferation are different from those for mycotoxin production, temperature (15.8–34.9°C) and humidity (34.2%–89.7%) ranges in the study sites were in the intervals reported as suitable for both microbial growth (Choi et al., 2015; Garbaba, Diriba, Ocho, & Hensel, 2018; Lane & Woloshuk, 2017) and mycotoxin production (Choi et al., 2015; Lane & Woloshuk, 2017; Murphy, Hendrich, Landgren, & Bryant, 2006) in cereals including rice. The optimum temperature for the growth of *Aspergillus* spp. has been reported to be 35°C while *Fusarium *spp. proliferation was fastest between 4 and 37°C (Mannaa & Kim, 2017; Sanchis & Magan, 2004).

### 3.2 Physical quality of rice and microbial load in study sites

**Figure 2:**
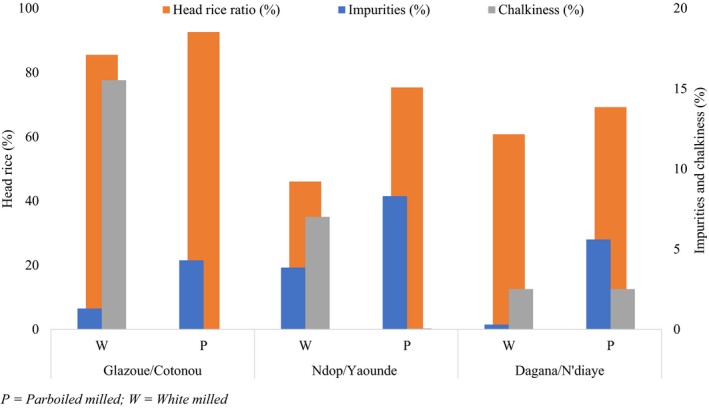
Selected physical grain quality characteristics of rice samples collected and stored at three sites in Africa and used for mycotoxin quantification

Physical grain qualities were evaluated in white and parboiled rice with the purpose of relating these qualities to mycotoxin levels at each site (Figure 2). The proportion of intact grains was higher in both parboiled and white milled rice, even though samples from Ndop‐Cameroon and Dagana‐Senegal recorded lower head rice ratios (46.1% and 60.8%, respectively) compared to those of Glazoue‐Benin (85.5%). Head rice is predominantly influenced by parboiling, drying, and milling (Buggenhout, Brijs, Celus, & Delcour, 2013). Chalkiness values in parboiled rice from Dagana were 2.5% while that for white rice was 15.5% in Glazoue, 7% in Ndop, and 2.5% in Dagana. Chalkiness is influenced by the genetic background of the variety (Gao et al., 2016), environmental conditions during the grain filling stage (Lanning, Siebenmorgen, Counce, Ambardekar, & Mauromoustakos, 2011; Mapiemfu et al., 2017), and the soaking and steaming regimes during parboiling (Graham‐Acquaah, Manful, Ndindeng, & Tchatcha, 2015; Zohoun, Ndindeng et al., 2018; Zohoun, Tang et al., 2018). In general, the level of impurities was higher in parboiled compared to white milled rice from all sites with the highest values recorded in parboiled samples from Ndop (8.3%). The level of impurities is influenced by harvesting, threshing, drying, parboiling, milling, and storage practices (AfricaRice, 2018). Previous studies have documented suboptimal pre‐ and postharvest practices in sites where samples for this study were collected (Mapiemfu et al., 2017; Ndindeng et al., 2015; Zohoun, Tang et al., 2018). The physical grain quality data recorded here are therefore akin to previous observations.

**Figure 3:**
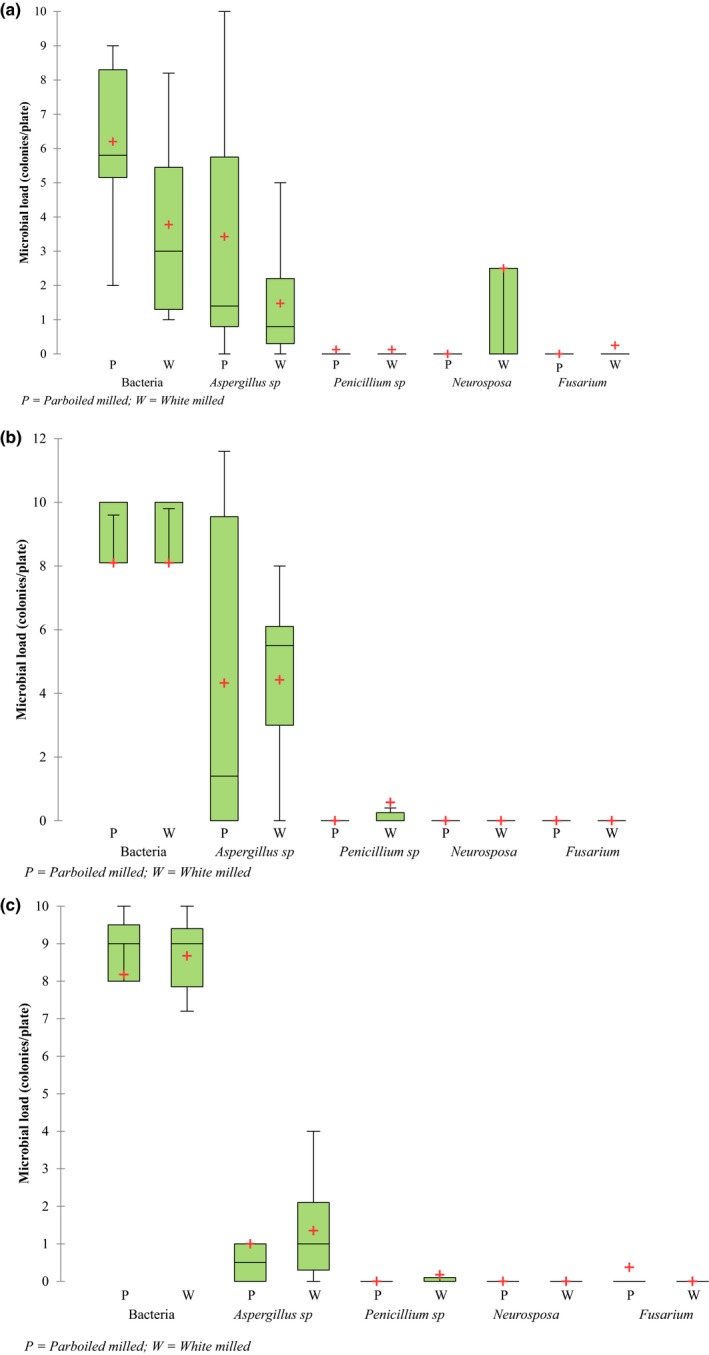
Combined microbial loads in zero‐ and 3‐month stored milled rice samples collected from (a) Glazoue and stored in Cotonou (Benin). (b) Ndop and stored in Yaoundé (Cameroon). (c) Dagana and stored in N'diaye (Senegal)

Different microbes including fungi and bacteria can invade rice from harvest to storage and even right to the table. The microbial load on white and parboiled milled rice evaluated at the start of storage and 3 months later is presented in Figure 3a–c respectively for samples collected/stored in Glazoue/Cotonou‐Benin, Ndop/Yaoundé‐Cameroon and Dagana/N'diaye‐Senegal. No difference was observed in the microbial load of BSS treated and untreated samples. Hence, the results on microbial loads reported here are those of the untreated samples only. Bacteria and the fungi (*Aspergillus*,* Penicillium*,* Neurospora*, and *Fusarium*) were identified at varying amounts in white and parboiled milled rice in all the sites and expressed as number of colonies per plate. Parboiled and white milled rice collected from the three sites were more susceptible to bacterial attack than all fungi. Bacteria represented the highest load in parboiled rice from Glazoué/Cotonou ranging from 2 to 9 colonies per plate, positively skewed with a median of 5.8 colonies. In white rice, most of the analyzed samples had bacteria ranging from 1 to 8.2, with a median of three colonies. White rice thus appears to be more susceptible to bacteria than parboiled rice, except for samples from Ndop/Yaoundé. In fact, parboiled and white rice from this location had similar and heavy bacterial loads ranging from 8.1 to 10, giving respective medians of 9.6 and 9.8 colonies. Bacterial load in white and parboiled milled rice from Dagana/N'diaye respectively varied between 8–10 and 7.2–10 colonies per plate with a common median of 9.00 colonies. Predominant bacteria associated with paddy rice in tropical environments are *Enterobacteriaceae*, *Bacillus* spp., *Pseudomonas* spp., *Xanthomonas* spp., *Cellulomonas flavigena,* and *Clavibacter michiganense* (Cottyn et al., 2001). Bacteria of pathogenic importance to humans characterized in rice are the spore‐forming *Bacillus cereus* and *Bacillus thuringiensis *(Ankolekar, Rahmati, & Labbé, 2009; Choi et al., 2014; Haque & Russell, 2005). Choi et al. (2014) observed that while mold and yeast population increased at temperature and relative humidity of 21°C and 85%, respectively; there was no increase in bacteria regardless of temperature and relative humidity. When paddy contaminated with heat resistant bacterial spore is soaked in water at initial temperature of 70–85°C as commonly practiced in artisanal parboiling systems, the bacterial populations are bound to increase. Bacterial spores resistant to heat during steaming will survive and be reactivated during drying and prolonged storage at room temperature (Ankolekar et al., 2009). This explains why bacterial loads were comparable in parboiled and white rice in the three sites. Based on this work and other reports (Magan & Aldred, 2007), it is suggested that farmers and processors observe proper hygiene during harvesting, threshing, drying, parboiling, milling, and storage and to regularly disinfect equipment to limit pathogenic bacteria in processed rice.

With regard to fungi, *Aspergillus* infested parboiled rice the most in samples from Glazoué/Cotonou. The number of *Aspergillus *spp. colonies per plate in parboiled rice from this site ranged from 0.3 to 10 with a median of 1.4 while in white rice, the median of *Aspergillus* identified was of 0.8 colonies. In samples collected/stored in Ndop/Yaoundé, *Aspergillus* spp. was abundant in parboiled rice with a range of 0–12 colonies per plate and a median of 1.4 colonies. In white rice from this site, *Aspergillus* spp. were concentrated between 3 and 6.1 colonies per plate and negatively skewed, with a median of 5.5 colonies. In samples from Dagana/N'diaye‐Senegal, *Aspergillus* spp. was more abundant in white than parboiled milled rice. *Penicillium* spp. were merely detected in white and parboiled rice from the three locations. Similar results were obtained for *Fusarium* and *Neurospora* spp., except for white rice from Glazoué/Cotonou from which a considerable number of *Neurospora* spp. colonies were isolated. Makun et al. (2007) identified a wide range of fungal species on stored moldy rice from Nigeria including over nine *Aspergillus* spp., four *Fusarium* spp., and four *Penicillium *spp. They also identified *Fusarium *spp. in field, stored, and marketed rice but *Neurospora* spp. was absent in the samples analyzed. Although *Fusarium* spp. occurred at low levels in this study, isolates of over ten *Fusarium* spp. among which *Fusarium proliferatum, Fusarium verticillioides,* and *Fusarium graminearum* were identified using molecular tools in diseased rice seed from Italy (Amatulli, Spadaroa, Gullinoa, & Garibaldia, 2010). In this study, *Aspergillus* spp. was the predominant genus occurring in both rice types and in all sites. Reddy et al. (2014) showed that in 675 stored paddy samples, *Aspergillus flavus* was the major mycofloral contaminant. *A. niger, A. parasiticus, A. ochraceus, A. terreus, A. versicolor, A. clavatus, A. fumigatus, *and *A. glaucus* have also been identified on rice and rice by‐products from field, stored, and marketed samples in different regions around the world (Makun et al., 2007; Sales & Yoshizawa, 2005; Reddy et al., 2014). *Penicillium* spp., another important storage fungus, was detected at low levels in only white rice from Benin and Senegal compared to the high incidence reported by Makun et al. (2007). *Neurospora* spp. detected at high levels in white rice from Benin is reported to be common on starchy‐rich foods and in subtropical and tropical regions (Davis & Perkins, 2002; Turner, Perkins, & Fairfield, 2001).

### 3.3 Modeling the effect of temperature, relative humidity, grain qualities, processing type, BSS treatment, and storage duration on FUM, ZEA, and AFLA concentrations in different sites

The concentrations of FUM, ZEA, and AFLA in relation to collection/storage location, rice processing type, BSS treatment, and storage duration are presented as Supporting Information S1. The effect of temperature, relative humidity, physical grain quality, sample collection/storage location, processing type and BSS treatment, and duration of storage in plastic woven bags on FUM, ZEA, and AFLA accumulation is shown in Table 2. The model's predictive power for all studied mycotoxins was robust (FUM [*p* < 0.0001; adjusted *R* ^2^ = 61.0%], ZEA [*p* < 0.0001; adjusted *R* ^2^ = 81.3%], and AFLA [*p* < 0.0001; adjusted *R* ^2^ = 52.3%]).

Fumonisin concentration was not affected by room storage temperature and relative humidity, physical grain quality (head rice yield, impurities, and chalkiness), processing type (data not shown), collection/storage location, and BSS treatment. However, storage duration had a positive effect on FUM concentration with samples stored at 0 and 3 months recording 0.50 and 0.37 ppm lower concentrations respectively compared to those stored for 6 months. This suggests that there is a high risk of FUM to accumulate to significant levels when rice is stored for 6 months and above in plastic woven bags in the studied sites. The range in FUM concentrations from 0.13 to 1.48 ppm across the three study sites was higher than the 0.4–4.4 and 132.5 ppb respectively quantified for FUM B1 and B2 by Makun et al. (2011). Although Abbas et al. (1998) did not detect FUMs in polished rice from cultivars in the Texas and Arkansas commercial rice fields in the USA, they quantified levels of 14.5, 1.2, 3.4, and 3.5 ppm in hulls, brown rice, bran, and unpolished rice respectively. The method used for quantifying these toxins was the competitive direct ELISA (CD–ELISA). In the same study using HPLC, higher accumulations of FUMs were reported. Sangare‐Tigori et al. (2006) and Ghali, Ghorbel, and Hedilli (2009) did not detect FUMs in ten and in eleven rice samples respectively from Ivorian and Tunisian markets. FUM levels reported in rice and rice products are generally low as observed in this study.

Zearalenone concentration was negatively influenced by the relative humidity in the storage rooms, head rice ratio but positively by the proportion of impurities. Parboiled samples recorded 85.4 ppm less ZEA compared to white milled samples (model table not shown). Sample collection/storage location also influences ZEA concentration with samples from Glazoue/Cotonou recording 400.3 ppm more ZEA compared with those from Dagana/N'diaye. Although samples from Ndop/Yaoundé recorded more ZEA (104.7 ppm) than those from Dagana/N'diaye, the difference was not significant. BSS treatment had no effect on ZEA concentration. Storage duration had a positive effect on ZEA concentration with samples stored at zero and three months recording 132.4 and 100.2 ppm lower concentrations respectively compared to those stored for 6 months. This suggests that the risk of ZEA accumulation was highest in rice samples with low head rice ratio, high proportions of impurities and stored for 6 months or above in plastic woven bags in the study sites. This risk was higher for samples collected/stored in Glazoue/Cotonou compared with those from Ndop/Yaoundé and Dagana/N'diaye. Using 1‐year‐old paddy collected from rice processing complexes in eight provinces of Korea, Lee et al. (2011) quantified ZEA levels ranging from 47–235, 26–3,156, and 25–3,305 ng/g (1 ng/g = 0.001 ppm) respectively in brown, blue‐tinged, and discolored rice samples. The authors did not find ZEA accumulation in polished rice. However, Sangare‐Tigori et al. (2006) quantified ZEA in polished rice with values ranging from 50 to 200 µg/kg (ppb) while Makun et al. (2011) quantified levels of 0–41.9 µg/kg.

Aflatoxin concentration was negatively affected by storage room temperature, head rice ratio and positively by proportion of impurities and chalky grains. AFLA concentration was also influenced by sample collection/storage locations with higher concentrations in samples collected/stored in Dagana/N'diaye and Glazoue/Cotonou compared with those from Ndop/Yaoundé. Parboiled samples recorded 4.02 ppm more AFLA compared to white milled samples (model table not shown). Like for FUM and ZEA, BSS treatment had no effect on AFLA concentration. Storage duration also had no effect on AFLA concentration. The above results suggest that the risk of AFLA accumulation was high in samples with low head rice ratio, high proportions of impurities, and chalky grains. This risk was lower for samples collected/stored in Ndop/Yaoundé compared with Dagana/N'diaye and Glazoue/Cotonou. The values of 1.03–45, 0.1–10.8, and 0.13–6.5 ppb respectively from Dagana/N'diaye, Glazoue/Cotonou, and Ndop/Yaoundé were lower than the 27.7–371.9 µg/kg (ppb) determined by HPLC in twenty‐one rice samples collected from the field, storage facilities, and markets in Nigeria (Makun et al., 2011). In Ivory Coast, Sangare‐Tigori et al. (2006) recorded a mean AFLA B1 concentration of 4.5 ppb in ten rice samples collected from local markets. On the contrary, Ghali, Khlifa, Ghorbel, Maaroufi, and Hedilli (2010) did not find AFLAs in eleven rice samples collected from domestic markets in Tunisia. These results indicate that the occurrence and concentration of AFLA are site‐specific with a strong dependence on production and processing practices rather than on storage methods. Therefore, routine screening of rice to be consumed by large segments of the population for AFLA is absolutely necessary. In the Asian continent where rice is most important in human nutrition and trade, *Aspergillus* contamination and occurrence of AFLAs in rice have been reported in some of the major exporters into West and Central Africa.

Temperatures of 33, 10–30, and 25–30°C have been reported as the optimum conditions to produce AFLAs (by *A. flavus* and *A. parasiticus*), FUM (by *F. verticillioides* and *F. proliferatum*), and ZEA (by *F. graminearum*), respectively (Murphy et al., 2006). Sorenson, Hesseltine, and Shotwell (1967) had reported an optimum temperature of 28°C for AFLA B1 and G1 production on rice by *Aspergillus flavus *and a comparable production of AFLA B1 at 32°C but not G1. Properly dried grains inside woven plastic bags under the temperature and relative humidity conditions reported in this study slowly equilibrated with the temperature and humidity conditions of the macroenvironment over the storage period. This observation supports previous findings by Williams, Baributsa, and Woloshuk (2001) that showed that at low relative humidity (<29%), grains stored in nylon woven bags but not in Purdue Improved Crop Storage (PICS) bags showed a decrease in moisture content.

The studied physical grain qualities (head rice, impurities, and chalkiness) had no effect on FUM concentration whereas ZEA and AFLA concentrations were negatively influenced by head rice but positively by impurities (Table 2). In addition, samples with higher chalky grains tended to record high AFLA concentrations. Magan and Aldred (2007) on the minimization of mycotoxin in the food chain underline mechanical damage and lack of hygiene during grain handling as critical factors that should be ultimately addressed to limit the contamination of maize by FUM and ZEA produced respectively by *F. proliferatum* and *F. graminearum*. AFLA contamination of Pakistani broken rice was reported to be higher than in whole white and brown rice (Iqbal, Asi, Ariño, Akram, & Zuber, 2012) for samples collected in the field (50%), processing units (42%), and retail markets (33%). Through histological and scanning electron microscopy, it is now known that the fungal invasion pattern of cereal grains follows intergranular spaces and/or damaged fissures from the pericarp through the aleurone to the endosperm (Ilag & Juliano, 1982; Jansen et al., 2005; Reddy, Reddy, Abbas, Abel, & Muralidharan, 2008; Windham, Williams, Mylroie, Reid, & Womack, 2018). This understanding explains the higher susceptibility of damaged grains (broken fractions) to fungi than intact grains. In addition to accessibility through cracks, pathogenic fungi produce extracellular hydrolytic enzymes that digest wax, cuticle, cell wall, and starch, facilitating grain invasion (Kikot, Alberto, & Alconada, 2009). This probably predisposes chalky rice whose endosperm is loose to AFLA producing fungi invasion than non chalky rice with a firm or hard endosperm as shown in this study. Oliveira et al. (2009) through correlations between *Fusarium* colonization and mechanical properties reached the conclusion that soft endosperm maize landraces from Brazil were highly susceptible to contamination. Expanding knowledge on the differences in fungal colonization of chalky and non chalky rice grains will be of great application in the rice value chain.

**Table 2:** Modeling the effect of storage room temperature and relative humidity, physical grain quality, burnt scallop shell treatment and storage duration on Fumonisin, Zearalenone, and Aflatoxin concentrations in samples collected/stored in three sites in Africa

	Source	[Fumonisin] (ppm)	[Zearalenone] (ppm)	[Aflatoxin] (ppb)
	Intercept	−0.57	1,265.9***	53.2**
Temperature and humidity in storage room	TEMP	0.04	−4.7	−1.8**
	RH	0.00	−5.3*	0.2
Physical grain quality	HR	0.00	−12.3***	−0.4**
	IMP	−0.01	19.7**	3.4***
	CLK	0.00	−2.0	0.7**
Collection/storage location	Glazoue/Cotonou	−0.23	400.3***	−3.9
	Ndop/Yaoundé	−0.03	104.7	−29.1***
	Dagana/N'diaye	—	—	—
BSS treatment	NT	0.04	6.1	0.4
	T	—	—	—
Duration of storage (months)	0	−0.50**	−132.4**	8.8
	3	−0.37**	−100.2**	2.3
	6	—	—	—
Model parameters	F	12.1	31.8	8.7
	Pr > F	<0.0001	<0.0001	<0.0001
	Adjusted *R* ^2^ (%)	61.0	81.3	52.3

Note

CLK: chalkiness; HR: head rice; IMP: impurities; NT: untreated; RH: relative humidity; T: treated; TEMP: temperature.

*** *p* < 0.0001.

** *p* < 0.001.

* *p* < 0.05; Dagana/N'diaye, treated and 6‐month duration were used as references in the model.

Although it was expected that parboiling will result in the control of fungi and mycotoxin in milled parboiled rice, Kaushik (2013) indicated that parboiling seemed not to be a favorable method for AFLA control. This view is further supported by Dors, Pinto, and Badiale‐Furlong (2009) who reported on the migration of mycotoxins from the outer layers into the endosperm during parboiling. In this study, parboiled samples recorded higher AFLA—confirming previous studies but lower ZEA concentrations compared to white milled rice suggesting a possible degradation of ZEA during parboiling.

In SSA, domestic milled rice (white and parboiled) is mostly of low quality and characterized by low head rice, high proportions of impurities, and chalky grains. In addition, both suboptimal pre‐ and postharvest practices are commonly used (Amponsah et al., 2017; Mapiemfu et al., 2017; Ndindeng et al., 2015). This rice is mostly sold in bulk or stored in plastic woven bags, jute bags, or bulk storage systems. In this study, the storage of grains in such systems was unsafe especially under Guinea savanna and Tropical forest climatic condition representing respectively subhumid and humid agroecological zones (HarvestChoice, 2009). Strategies to reduce the risk of mycotoxin contamination in SSA will include improvement of the physical rice quality (head rice ratio, proportion of impurities, and chalky grain). This can be achieved through the use of improved pre‐ and postharvest practices and proper packaging of rice in hermetic systems before marketing and storage at higher than ambient temperatures. Although relative humidity and temperature did not affect ZEA and FUM accumulation in this study, Choi et al. (2015) reported temperature and relative humidity of 21°C and 97% respectively, as suitable for *Fusarium *proliferation and mycotoxin production.

Burnt scallop shell powder neither affected bacterial load nor mycotoxin concentration in this study. However, bactericidal action of heated scallop shell powder (CaO) against *Psuedomonas aeruginosa* biofilms on eggshell has been reported at 0.05%–0.3% concentration (Jung et al., 2017). It is suspected that the 0.1% concentration of the BSS powder used in this study was insufficient because the storage system freely allowed the exchange of moisture between the stored samples and the environment. Thus, the need to further investigate the efficacy of various concentrations and dose application rate for the control of mycotoxins in stored grains using different storage systems.

## 4 CONCLUSIONS

The environmental conditions of temperature and relative humidity in the Guinea savanna (subhumid), Tropical forest (humid), and Sahel (semi‐arid) zones predisposes rice stored in plastic woven or jute bags to moisture re‐absorption (re‐wetting) or moisture loss (drying), thus compromising its quality during storage. Therefore, rice should be stored in hermetic systems especially for periods longer than 3 months. Poor physical qualities (low head rice, high proportions of impurities, and chalkiness) of rice produced in SSA enhance microbial colonization and consequently mycotoxin accumulation. Processing (white and parboiled milled rice production) affected mycotoxin concentration differently as AFLA concentration was higher in parboiled samples while ZEA concentration was higher in white milled samples. Storage duration affected mycotoxin concentration differently as FUM and ZEA concentration increased with duration of storage while AFLA did not. The 0.1% concentration of the BSS powder used did not affect the microbial load and total FUM, ZEA, and AFLA concentrations. The BSS dose used was insufficient because the storage system facilitated the exchange of moisture with the environment. Strategies to reduce the risk of mycotoxin contamination in those sites will encompass the improvement of the physical rice qualities (head rice ratio, impurities, and chalky grain) through better pre‐ and postharvest practices and proper packaging of both treated and untreated rice in hermetic systems prior to marketing and/or storage.

## CONFLICT OF INTEREST

The authors declare that they do not have any conflict of interest.

## ETHICAL REVIEW

This study does not involve any human or animal testing.

## References

[fsn3959-bib-0001] Abbas, H. , Cartwright, R. , Shier, W. , Abouzied, M. , Rice, L. , Ross, P. , … Meredith, F. (1998). Natural occurrence of fumonisins in rice with Fusarium sheath rot disease. Plant Disease, 82, 22–25. 10.1094/pdis.1998.82.1.22 30857062

[fsn3959-bib-0002] Abia, W. A. , Warth, B. , Sulyok, M. , Krska, R. , Tchana, A. N. , Njobeh, P. B. , … Moundipa, P. F. (2013). Determination of multi‐mycotoxin occurrence in cereals, nuts and their products in Cameroon by liquid chromatography tandem mass spectrometry (LC‐MS/MS). Food Control, 31, 438–453. 10.1016/j.foodcont.2012.10.006

[fsn3959-bib-0003] Africa Rice Center (AfricaRice) (2018). Africa Rice Center (AfricaRice) Annual Report 2017: More effective targeting of research for development. Abidjan, Côte d’Ivoire: 40 pp.

[fsn3959-bib-0004] Almeida, M. I. , Almeida, N. G. , Carvalho, K. L. , Gonçalves, G. A. A. , Silva, C. N. , Santos, E. A. , … Vargas, E. A. (2012). Co‐occurrence of aflatoxins B1, B2, G1 and G2, ochratoxin A, zearalenone, deoxynivalenol, and citreoviridin in rice in Brazil. Food Additive and Contaminants, 29, 694–703. 10.1080/19440049.2011.651750 22316345

[fsn3959-bib-0005] Amatulli, T. M. , Spadaroa, D. , Gullinoa, L. M. , & Garibaldia, A. (2010). Molecular identification of Fusarium spp. associated with bakanae disease of rice in Italy and assessment of their pathogenicity. Plant Pathology, 59, 839–844. 10.1111/j.1365-3059.2010.02319.x

[fsn3959-bib-0006] Amponsah, S. K. , Addo, A. , Dzisi, K. A. , Moreira, J. , & Ndindeng, S. A. (2017). Performance evaluation and field characterization of the sifang mini rice combine harvester. Applied Engineering in Agriculture, 33, 479–489. 10.13031/aea.11876

[fsn3959-bib-0007] Ankolekar, C. , Rahmati, T. , & Labbé, G. R. (2009). Detection of toxigenic Bacillus cereus and Bacillus thuringiensis spores in U.S. rice. International Journal of Food Microbiology, 128, 460–466. 10.1016/j.ijfoodmicro.2008.10.006 19027973

[fsn3959-bib-0008] Audenaert, K. , Vanheule, A. , Höfte, M. , & Haesaert, G. (2014). Deoxynivalenol: A major player in the multifaceted response of Fusarium to its environment. Toxins, 6, 1274–19. 10.3390/toxins6010001 24451843 PMC3920246

[fsn3959-bib-0009] Bandara, J. M. , Vithanege, A. K. , & Bean, G. A. (1991). Occurrence of aflatoxins in parboiled rice in Sri Lanka. Mycopathologia, 116, 1485–1487. 10.1007/bf00436366 1779999

[fsn3959-bib-0010] Barnett, H. L. , & Hunter, B. B. (1972). Illustrated genera of imperfect fungi (p. 241). Minneapolis, MN: Burgess publishing Co.

[fsn3959-bib-0011] Bensassi, F. , Zaied, C. , Abid, S. , Hajlaoui, M. R. , & Bacha, H. (2010). Occurrence of deoxynivalenol in durum wheat in Tunisia. Food Control, 21, 281–285. 10.1016/j.foodcont.2009.06.005

[fsn3959-bib-0012] Buggenhout, J. , Brijs, K. , Celus, I. , & Delcour, J. A. (2013). The breakage susceptibility of raw and parboiled rice: A review. Journal of Food Engineering, 117, 304–315. 10.1016/j.jfoodeng.2013.03.009

[fsn3959-bib-0013] Center for Disease Control (CDC) (2004). Outbreak of aflatoxin poisoning – Eastern and central provinces, Kenya, January–July. Morbidity and Mortality Weekly Report. 53, 790–3. Retrieved from http://www.cdc.gov/nceh/hsb/chemicals/mmwr-aflatoxin.pdf 15343146

[fsn3959-bib-0014] Cheli, F. , Pinotti, L. , Rossi, L. , & Dell’Orto, V. (2013). Effect of milling procedures on mycotoxin distribution in wheat fractions: A review. LWT – Food Science and Technology, 54, 307–314. 10.1016/j.lwt.2013.05.040

[fsn3959-bib-0015] Choi, S. , Kim, H. , Kim, Y. , Kim, B.‐s. , Beuchat, R. L. , & Ryu, H. J. (2014). Fate of Bacillus cereus and naturally occurring microbiota on milled rice as affected by temperature and relative humidity. Food Microbiology, 38, 122–127. 10.1016/j.fm.2013.08.016 24290634

[fsn3959-bib-0016] Choi, S. , Jun, H. , Bang, J. , Chung, S.‐H. , Kim, Y. , Kim, B. , … Ryu, J.‐H. (2015). Behaviour of Aspergillus flavus and *Fusarium graminearum* on rice as affected by degree of milling, temperature, and relative humidity during storage. Food Microbiology, 46, 307–313. 10.1016/j.fm.2014.08.019 25475300

[fsn3959-bib-0017] Christensen, C. M. , & Kaufmann, H. H. (1965). Deterioration of stored grains by fungi. Annual Review of Phytopathology, 3, 69–84. 10.1146/annurev.py.03.090165.000441

[fsn3959-bib-0018] Cottyn, B. , Regalado, E. , Lanoot, B. , Cleene, M. D. , Mew, T. W. , & Swings, J. (2001). Bacterial populations associated with rice seed in the tropical environment. Phytopathology, 91, 282–292. 10.1094/phyto.2001.91.3.282 18943348

[fsn3959-bib-0019] Davis, R. H. , & Perkins, D. D. (2002). Neurospora: A model of model microbes. Nature Reviews Genetics, 3, 397–403. 10.1038/nrg797 11988765

[fsn3959-bib-0020] Dors, G. C. , Pinto, L. A. A. , & Badiale‐Furlong, E. (2009). Migration of mycotoxins into rice starchy endosperm during the parboiling process. LWT – Food Science and Technology, 42, 433–437. 10.1016/j.lwt.2008.03.012

[fsn3959-bib-0021] Dossou, B. , & Silue, D. (2018). Rice pathogens intercepted on seeds originating from 11 African countries and from the USA. Seed Science and Technology, 46(1), 31–40. 10.15258/sst.2018.46.1.03

[fsn3959-bib-0022] Fleurat‐Lessard, F. (2017). Integrated management of the risks of stored grain spoilage by seedborne fungi and contamination by storage mould mycotoxins – An update. Journal of Stored Product Research, 71, 22–40. 10.1016/j.jspr.2016.10.002

[fsn3959-bib-0023] Gaag, B. V. D. , Spath, S. , Dietrich, H. , Stigter, E. , Boonzaaijer, G. , van Osenbruggen, T. , & Koopal, K. (2003). Biosensors and multiple mycotoxin analysis. Food Control, 14, 251–254. 10.1016/s0956-7135(03)00008-2

[fsn3959-bib-0024] Gao, Y. , Liu, C. , Li, Y. , Zhang, A. , Dong, G. , Xie, L. , … Gao, Z. (2016). QTL analysis for chalkiness of rice and fine mapping of a candidate gene for qACE9. Rice, 9, 41 10.1186/s12284-016-0114-5 27549111 PMC4993740

[fsn3959-bib-0025] Garbaba, A. C. , Diriba, S. , Ocho, L. F. , & Hensel, O. (2018). Potential for mycotoxin‐producing fungal growth in various agro‐ecological settings and maize storage systems in southwestern Ethiopia. Journal of Stored Products Research, 76, 22–29. 10.1016/j.jspr.2017.12.001

[fsn3959-bib-0026] Gelderblom, W. C. , Jaskiewicz, K. , Marasas, W. F. O. , Thiel, P. G. , Horak, R. M. , Vleggaar, R. , & Kriek, N. P. J. (1988). Fumonisins—Novel mycotoxins with cancer promoting activity produced by Fusarium moniliforme. Applied and Environmental Microbiology, 54, 1806–1811. 2901247 10.1128/aem.54.7.1806-1811.1988 PMC202749

[fsn3959-bib-0027] Gelderblom, W. , Kriek, N. , Marasas, W. , & Thiel, P. (1991). Toxicity and carcinogenicity of the Fusarium moniliforme metabolite, fumonisin B1, in rats. Carcinogenesis, 12, 1247–1251. 10.1093/carcin/12.7.1247 1649015

[fsn3959-bib-0028] Gelderblom, W. , Marasas, W. , Lebepe‐Mazur, S. , Swanevelder, S. , Vessey, C. , & de la M Hall, P. (2002). Interaction of fumonisin B1 and aflatoxin B1 in a short‐term carcinogenesis model in rat liver. Toxicology, 171, 161–173. 10.1016/s0300-483x(01)00573-x 11836022

[fsn3959-bib-0029] Ghali, R. , Ghorbel, H. , & Hedilli, A. (2009). Fumonisin determination in Tunisian foods and feeds. ELISA and HPLC methods comparison. Journal of the Science of Food and Agriculture, 57, 3955–3960. 10.1021/jf803786h 19298079

[fsn3959-bib-0030] Ghali, R. , Hmaissia‐Khlifa, K. , Ghorbel, H. , Maaroufi, K. , & Hedili, A. (2008). Incidence of aflatoxins, ochratoxin A and zearalenone in Tunisian foods. Food Control, 19, 921–924. 10.1016/j.foodcont.2007.09.003

[fsn3959-bib-0031] Ghali, R. , Khlifa, H. K. , Ghorbel, H. , Maaroufi, K. , & Hedilli, A. (2010). Aflatoxin determination in commonly consumed foods in Tunisia. Journal of the Science of Food and Agriculture, 90, 2347–2351. 10.1002/jsfa.4069 20812375

[fsn3959-bib-0032] Graham‐Acquaah, S. , Manful, J. T. , Ndindeng, S. A. , & Tchatcha, D. A. (2015). Effect of soaking and steaming regimes on the quality of artisanal parboiled rice. Journal of Food Processing and Preservation, 39, 2286–2296. 10.1111/jfpp.12474

[fsn3959-bib-0033] Gummert, M. , Balingbing, C. B. , Barry, G. , & Estevez, L. A. (2009). Management options, technologies and strategies for minimised mycotoxin contamination of rice. World Mycotoxin Journal, 2, 151–159. 10.3920/wmj2008.1131

[fsn3959-bib-0034] Haque, A. , & Russell, N. J. (2005). Phenotypic and genotypic characterization of Bacillus cereus isolates from Bangladeshi rice. International Journal of Food Microbiology, 98, 23–34. 10.1016/j.ijfoodmicro.2004.04.025 15617798

[fsn3959-bib-0035] HarvestChoice (2009). AEZ Tropical (5‐class). International Food Policy Research Institute, Washington, DC, and University of Minnesota, St. Paul, MN.

[fsn3959-bib-0036] Ilag, L. L. , & Juliano, B. O. (1982). Colonisation and aflatoxin formation by Aspergillus spp. on brown rices differing in endosperm properties. Journal of the Science of Food and Agriculture, 33, 97–102. 10.1002/jsfa.2740330117 6803064

[fsn3959-bib-0037] International Agency for Research on Cancer (1993). Aflatoxins: Naturally occurring aflatoxins (Group 1), aflatoxins M1 (Group 2B). International Agency for Research on Cancer, 56, 245.

[fsn3959-bib-0038] Iqbal, S. Z. , Asi, M. R. , Ariño, A. , Akram, N. , & Zuber, M. (2012). Aflatoxin contamination in different fractions of rice from Pakistan and estimation of dietary intakes. Mycotoxin Research, 28(3), 175–180. 10.1007/s12550-012-0131-1 23606125

[fsn3959-bib-0039] Iqbal, S. Z. , Jinap, S. , Pirouz, A. A. , & Ahmad Faizal, A. R. (2015). Aflatoxin M1 in milk and dairy products, occurrence and recent challenges: A review. Trends in Food Science Technology, 46, 110–119. 10.1016/j.tifs.2015.08.005

[fsn3959-bib-0040] Jansen, C. , von Wettstein, D. , Schäfer, W. , Kogel, K. H. , Felk, A. , & Maier, F. J. (2005). Infection patterns in barley and wheat spikes inoculated with wild‐type and trichothecene synthase gene disrupted *Fusarium graminearum* . Proceedings of the National Academy of Sciences of USA, 102, 16892–16897. 10.1073/pnas.0508467102 PMC1283850 16263921

[fsn3959-bib-0041] Juan, C. , Zinedine, A. , Idrissi, L. , & Mañes, J. (2008). Ochratoxin A in rice on the Moroccan retail market. International Journal of Food Microbiology, 126, 83–85. 10.1016/j.ijfoodmicro.2008.05.005 18550188

[fsn3959-bib-0042] Jung, J. , Shin, S. , Park, Y. , Kim, E. S. , Kang, I. , Park, J. , … Ha, D. S. (2017). Bactericidal effect of calcium oxide (Scallop‐Shell Powder) against *Pseudomonas aeruginosa* biofilm on quail egg shell, stainless steel, plastic, and rubber. Journal of Food Science, 82, 1682–1687. 10.1111/1750-3841.13753 28627772

[fsn3959-bib-0043] Kaushik, G. (2013). Effect of processing on mycotoxin content in grains. Critical Reviews in Food Science and Nutrition, 55, 1672–1683. 10.1080/10408398.2012.701254 24915313

[fsn3959-bib-0044] Kikot, E. G. , Alberto, R. H. , & Alconada, M. T. (2009). Contribution of cell wall degrading enzymes to pathogenesis of *Fusarium graminearum*: A review. Journal of Basic Microbiology, 49, 231–241. 10.1002/jobm.200800231 19025875

[fsn3959-bib-0045] Kini, K. , Agnimonhan, R. , Afolabi, O. , Soglonou, B. , Silué, D. , & Koebnik, R. (2017b). First report of a new bacterial leaf blight of rice caused by *Pantoea ananatis* and *Pantoea stewartii* in Togo. Plant Disease, 101, 241 10.1094/pdis-06-16-0939-pdn

[fsn3959-bib-0046] Kini, K. , Agnimonhan, R. , Afolabi, O. , Milan, B. , Soglonou, B. , Gbogbo, V. , … Silué, D. (2017a). First report of a new bacterial leaf blight of rice caused by *Pantoea ananatis* and *Pantoea stewartii* in Benin. Plant Disease, 101, 242 10.1094/pdis-06-16-0940-pdn

[fsn3959-bib-0047] Kini, K. , Agnimonhan, R. , Dossa, R. , Soglonou, B. , Gbogbo, V. , Ouedraogo, I. , … Silue, D. (2017c). First report of *Sphingomonas* sp. causing bacterial leaf blight of rice in Benin, Burkina Faso, The Gambia, Ivory Coast, Mali, Nigeria, Tanzania and Togo. New Disease Reports, 35, 32 10.5197/j.2044-0588.2017.035.032

[fsn3959-bib-0048] Kostro, K. , Gajęcka, M. , Lisiecka, U. , Majer‐Dziedzic, B. , Obremski, K. , Zielonka, L. , & Gajęcki, M. (2011). Subpopulation of lymphocytes CD4+ and CD8+ in peripheral blood of sheep with zearalenone mycotoxicosis. Bulletin of the Veterinary Institute in Pulawy, 55, 241–246.

[fsn3959-bib-0049] Lane, B. , & Woloshuk, C. (2017). Impact of storage environment on the efficacy of hermetic storage bags. Journal of Stored Products Research, 72, 83–89. 10.1016/j.jspr.2017.03.008 28659648 PMC5476195

[fsn3959-bib-0050] Lang, J. M. , Hamilton, J. P. , Diaz, M. G. Q. , Sluys, M. A. V. , Burgos, M. R. G. , Cruz, C. M. V. , … Leach, J. E. (2010). Genomics‐based diagnostic marker development for *Xanthomonas oryzae *pv. *oryzae *and *X. oryzae *pv. *oryzicola* . Plant Disease, 94, 311–319. 10.1094/pdis-94-3-0311 30754246

[fsn3959-bib-0051] Lanning, S. B. , Siebenmorgen, T. J. , Counce, P. A. , Ambardekar, A. A. , & Mauromoustakos, A. (2011). Extreme nighttime air temperatures in 2010 impact rice chalkiness and milling quality. Field Crops Research, 124, 132–136. 10.1016/j.fcr.2011.06.012

[fsn3959-bib-0052] Lee, H. J. , & Ryu, D. (2017). Worldwide occurrence of mycotoxins in cereals and cereal derived food products: Public health perspectives of their co‐occurrence. Journal of Agricultural and Food Chemistry, 65, 7034–7051. 10.1021/acs.jafc.6b04847 27976878

[fsn3959-bib-0053] Lee, T. , Lee, S. H. , Lee, S. H. , Shin, Y. J. , Yun, J.‐C. , Lee, Y.‐W. , & Ryu, J.‐G. (2011). Occurrence of Fusarium mycotoxins in rice and its milling by‐products in Korea. Journal of Food Protection, 74, 1169–1174. 10.4315/0362-028x.jfp-10-564 21740720

[fsn3959-bib-0054] Magan, N. , & Aldred, D. (2007). Post‐harvest control strategies: Minimizing mycotoxins in the food chain. International Journal of Food Microbiology, 119, 131–139. 10.1016/j.ijfoodmicro.2007.07.034 17764773

[fsn3959-bib-0055] Magan, N. , Hope, R. , Cairns, V. , & Aldred, D. (2003). Post‐harvest fungal ecology: Impact of fungal growth and mycotoxin accumulation in stored grain. European Journal of Plant Pathology, 9, 723–730. 10.1007/978-94-017-1452-5_7

[fsn3959-bib-0056] Magan, N. , Medina, A. , & Aldred, D. (2011). Possible climate‐change effects on mycotoxin contamination of food crops pre‐ and postharvest. Plant Pathology, 60, 150–163. 10.1111/j.1365-3059.2010.02412.x

[fsn3959-bib-0057] Majumder, S. , Bala, B. K. , Arshad, M. F. , Haque, A. M. , & Hossain, A. M. (2016). Food security through increasing technical efficiency and reducing postharvest losses of rice production systems in Bangladesh. Food Security, 8, 361–374. 10.1007/s12571-016-0558-x

[fsn3959-bib-0058] Makun, H. A. , Dutton, M. F. , Njobeh, P. B. , Mwanza, M. , & Kabiru, A. Y. (2011). Natural multi‐occurrence of mycotoxins in rice from Niger State, Nigeria. Mycotoxin Research, 27, 97–104. 10.1007/s12550-010-0080-5 21836766 PMC3150825

[fsn3959-bib-0059] Makun, H. A. , Gbodi, T. A. , Akanya, O. H. , Salako, E. A. , & Ogbadu, G. H. (2007). Fungi and some mycotoxins contaminating rice (*Oryza sativa*) in Niger state, Nigeria. African Journal of Biotechnology, 6, 99–108.

[fsn3959-bib-0060] Mannaa, M. , & Kim, D. K. (2017). Influence of temperature and water activity on deleterious fungi and mycotoxin production during grain storage. Mycobiology, 45, 240–254. 10.5941/myco.2017.45.4.240 29371792 PMC5780356

[fsn3959-bib-0061] Mapiemfu, D. L. , Ndindeng, S. A. , Ambang, Z. , Tang, E. N. , Ngome, F. , Johnson, J. M. , … Saito, K. (2017). Physical rice grain quality as affected by biophysical factors and pre‐harvest practices. International Journal of Plant Production, 11, 561–576. https://doi.org/10.22069ijpp.2017.3718.

[fsn3959-bib-0062] Matumba, L. , Monjerezi, M. , Khonga, E. B. , & Lakudzala, D. D. (2011). Aflatoxins in sorghum, sorghum malt and traditional opaque beer in southern Malawi. Food Control, 22, 266–268. 10.1016/j.foodcont.2010.07.008

[fsn3959-bib-0063] McClenny, N. (2005). Laboratory detection and identification of *Aspergillus* species by microscopic observation and culture: The traditional approach. Medical Mycology, 43(Suppl. 1), S125–S128. 10.1080/13693780500052222 16110804

[fsn3959-bib-0064] Meky, F. , Hardie, L. , Evans, S. , & Wild, C. (2001). Deoxynivalenol‐induced immunomodulation of human lymphocyte proliferation and cytokine production. Food and Chemical Toxicology, 39, 827–836. 10.1016/s0278-6915(01)00029-1 11434990

[fsn3959-bib-0065] Murphy, P. A. , Hendrich, S. , Landgren, C. , & Bryant, M. C. (2006). Food mycotoxins: An update. Journal of Food Science, 7, R51–R65. 10.1111/j.1750-3841.2006.00052.x

[fsn3959-bib-0066] Muthomi, J. W. , Ndung’u, J. K. , Gathumbi, J. K. , Mutitu, E. W. , & Wagacha, J. M. (2008). The occurrence of Fusarium species and mycotoxins in Kenyan wheat. Crop Protection, 27, 1215–1219. 10.1016/j.cropro.2008.03.001

[fsn3959-bib-0067] Ndindeng, S. A. , Manful, J. , Futakuchi, K. , Mapiemfu‐Lamare, D. , Akoa‐Etoa, J. M. , Tang, E. N. , … Moreira, J. (2015). Upgrading the quality of Africa’s rice: A novel artisanal parboiling technology for rice processors in sub‐Saharan Africa. Food Science and Nutrition, 3, 557–568. 10.1002/fsn3.242 26788297 PMC4708646

[fsn3959-bib-0068] Nelson, P. E. , Toussoun, T. A. , & Marasas, W. F. O. (1983). Fusarium species: An illustrated manual for identification. University Park, PA: The Pennsylvania State University Press.

[fsn3959-bib-0069] Njobeh, P. B. , Dutton, M. F. , Koch, S. H. , Chuturgoon, A. A. , Stoev, S. D. , & Mosonik, J. S. (2010). Simultaneous occurrence of mycotoxins in human food commodities from Cameroon. Mycotoxin Research, 26, 47–57. 10.1007/s12550-009-0039-6 23605240

[fsn3959-bib-0070] Oliveira, T. R. , Jaccoud‐Filho, D. S. , Henneberg, L. , Michel, M. D. , Demiate, I. M. , Pinto, A. T. B. , … Barana, A. C. (2009). Maize (Zea mays L.) landraces from the southern region of Brazil: Contamination by Fusarium sp., zearalenone, physical and mechanical characteristics of the kernels. Brazilian Archives of Biology and Technology, 52, 11–16. 10.1590/s1516-89132009000700002

[fsn3959-bib-0071] Pitt, J. I. , & Hocking, A. D. (1997). Fungi and food spoilage. London, UK: Blackie Academic and Professional.

[fsn3959-bib-0072] Reddy, K. R. N. , Reddy, C. S. , & Muralidharan, K. (2009). Detection of *Aspergillus* spp. and aflatoxin B1 in rice in India. Food Microbiology, 26, 27–31. 10.1016/j.fm.2008.07.013 19028301

[fsn3959-bib-0073] Reddy, N. R. K. , Reddy, S. C. , Abbas, K. H. , Abel, A. C. , & Muralidharan, K. (2008). Mycotoxigenic fungi, mycotoxins, and management of rice grains. Toxin Reviews, 27, 287–317. 10.1080/15569540802432308

[fsn3959-bib-0074] Sales, A. , & Yoshizawa, T. (2005). Updated profile of aflatoxin and Aspergillus section Flavi contamination in rice and its by‐products from the Philippines. Food Additives and Contaminants, 22, 429–436. 10.1080/02652030500058387 16019814

[fsn3959-bib-0075] Sanchis, V. , & Magan, N. (2004). Environmental profiles for growth and mycotoxin production In Magan N., & Olsen M. (Eds.), Mycotoxins in food: Detection and control (pp. 174–189). Cambridge, UK: Woodhead Publishing Ltd.

[fsn3959-bib-0076] Sangare‐Tigori, B. , Moukha, S. , Kouadio, H. J. , Betbeder, A. M. , Dano, D. S. , & Creppy, E. E. (2006). Co‐occurrence of aflatoxin B1, fumonisin B1, ochratoxin A and zearalenone in cereals and peanuts from Côte d’Ivoire. Food Additives and Contaminants, 23, 1000–1007. 10.1080/02652030500415686 16982522

[fsn3959-bib-0077] Sheahan, M. , & Barrett, B. C. (2017). Review: Food loss and waste in Sub‐Saharan Africa. Food Policy, 70, 1274–12. 10.1016/j.foodpol.2017.03.012 PMC5555439 28839345

[fsn3959-bib-0078] Sorenson, W. G. , Hesseltine, C. W. , & Shotwell, O. L. (1967). Effect of temperature on production of aflatoxin on rice by Aspergillus flavus. MycopathologiaEt Mycologia Applicata, 33, 49–55. 10.1007/bf02049790

[fsn3959-bib-0079] Turner, B. C. , Perkins, D. D. , & Fairfield, A. (2001). Neurospora from natural populations: A global study. Fungal Genetics and Biology, 32, 67–92. 10.1006/fgbi.2001.1247 11352529

[fsn3959-bib-0080] Williams, J. , Phillips, T. D. , Jolly, P. E. , Stiles, J. K. , Jolly, C. M. , & Aggarwal, D. (2004). Human aflatoxicosis in developing countries: A review of toxicology, exposure, potential health consequences, and interventions. American Journal of Clinical Nutrition, 80, 1106–1122. 10.1093/ajcn/80.5.1106 15531656

[fsn3959-bib-0081] Williams, S. B. , Baributsa, D. , & Woloshuk, C. (2014). Assessing Purdue Improved Crop Storage (PICS) bags to mitigate fungal growth and aflatoxin contamination. Journal of Stored Products Research, 59, 190–196. 10.1016/j.jspr.2014.08.003

[fsn3959-bib-0082] Windham, G. L. , Williams, P. W. , Mylroie, J. E. , Reid, C. X. , & Womack, E. D. (2018). A histological study of Aspergillus flavus colonization of wound inoculated maize kernels of resistant and susceptible maize hybrids in the field. Frontiers in Microbiology, 9, 10.3389/fmicb.2018.00799 PMC5928334 29740423

[fsn3959-bib-0083] Zaied, C. , Abid, S. , Zorgui, L. , Bouaziz, C. , Chouchane, S. , Jomaa, M. , & Bacha, H. (2009). Natural occurrence of ochratoxin A in Tunisian cereals. Food Control, 20, 218–222. 10.1016/j.foodcont.2008.05.002

[fsn3959-bib-0084] Zaied, C. , Zouaoui, N. , Bacha, H. , & Abid, S. (2012). Natural occurrence of zearalenone in Tunisian wheat grains. Food Control, 25, 773–777. 10.1016/j.foodcont.2011.12.012

[fsn3959-bib-0085] Zinedine, A. , Brera, C. , Elakhdari, S. , Catano, C. , Debegnach, F. , Angelini, S. , … Minardi, V. (2006). Natural occurrence of mycotoxins in cereals and spices commercialized in Morocco. Food Control, 17, 868–874. 10.1016/j.foodcont.2005.06.001

[fsn3959-bib-0086] Zinedine, A. , Soriano, J. M. , Juan, C. , Mojemmi, B. , Molto, J. C. , Bouklouze, A. , … Manes, J. (2007). Incidence of ochratoxin A in rice and dried fruits from Rabat and Sale area, Morocco. Food Additives and Contaminants, 24, 285–291. 10.1080/02652030600967230 17364931

[fsn3959-bib-0087] Zohoun, E. V. , Ndindeng, S. A. , Soumanou, M. M. , Tang, E. N. , Bigoga, J. , Manful, J. , … Futakuchi, K. (2018b). Appropriate parboiling steaming time at atmospheric pressure and variety to produce rice with weak digestive properties. Food Science and Nutrition, 6, 757–764. 10.1002/fsn3.617 29983937 PMC6021733

[fsn3959-bib-0088] Zohoun, E. V. , Tang, E. N. , Soumanou, M. M. , Manful, J. , Akissoe, N. H. , Bigoga, J. , … Ndindeng, S. A. (2018a). Physicochemical and nutritional properties of rice as affected by parboiling steaming time at atmospheric pressure and variety. Food Science and Nutrition, 6, 638–652. 10.1002/fsn3.600 29876115 PMC5980200

