# Supplementary material for: Mycotoxin concentrations in rice from three climatic locations in Africa as affected by grain quality, production site, and storage duration
Source: Food Sci Nutr. 2019 Feb 11;7(4):1274–87. doi: 10.1002/fsn3.959 (PMC6475755; doi:10.1002/fsn3.959)
Supplement: Supplementary file 1 [file FSN3-7-1274-s001.docx]

**Supplementary information 1: Fumonisin, zearalenone and aflatoxin concentrations by collection/ storage site, processing type, burnt scallop shell treatment and storage duration.**

| Factor | variable | Obs. | [Fumonisin]  (ppm) | | | [Zearalenone]  (ppm) | | | [Aflatoxin]  (ppb) | | |
| --- | --- | --- | --- | --- | --- | --- | --- | --- | --- | --- | --- |
|  |  |  | Min–Max | mean | Std. Dev. | Min–Max | mean | Std. Dev. | Min–Max | mean | Std. Dev. |
| Collection/storage location | Glazoue/Cotonou | 24 | 0.13–0.91 | 0.37 | 0.21 | 0.16–36.72 | 6.22 | 9.60 | 0.05–10.78 | 3.05 | 2.78 |
|  | Ndop/Yaoundé | 24 | 0.14–0.94 | 0.39 | 0.23 | 0.25–511.58 | 133.25 | 162.37 | 0.13–6.46 | 1.64 | 1.44 |
|  | Dagana/N’diaye | 24 | 0.16–1.48 | 0.48 | 0.35 | 0.10–14.73 | 3.09 | 4.41 | 1.03–45.00 | 9.80 | 12.06 |
| Kruskal Wallis test | | | Chi-square =1.48, df = 2, p = 0.47 | | | Chi-square = 17.34, df = 2, p < 0.0001 | | | Chi-square = 14.94, df = 2, p = 0.001 | | |
| Processing type | P | 36 | 0.13–1.48 | 0.43 | 0.29 | 0.10–36.27 | 5.26 | 8.35 | 0.05–44.99 | 6.84 | 10.70 |
|  | W | 36 | 0.13–1.18 | 0.40 | 0.26 | 0.16–511.58 | 89.78 | 145.66 | 0.13–10.78 | 2.82 | 2.32 |
| Mann Whitney U test | | | U = 599.0, p = 0.58 | | | U = 485.5, p = 0.06 | | | U = 628.0, p = 0.82 | | |
| BSS treatment | NT | 36 | 0.14–1.18 | 0.43 | 0.25 | 0.25–511.58 | 50.58 | 120.91 | 0.13–36.62 | 5.01 | 8.09 |
|  | T | 36 | 0.13–1.48 | 0.40 | 0.29 | 0.10–403.33 | 44.47 | 101.60 | 0.05–45.00 | 4.65 | 7.91 |
| Kruskal Wallis test | | | U = 548.0, p = 0.26 | | | U = 562, P = 0.33 | | | U = 635.5, P = 0.88 | | |
| Duration of storage  (months) | 6 | 24 | 0.32–1.48 | 0.70 | 0.26 | 2.95–511.58 | 83.00 | 161.39 | 0.61–17.04 | 4.12 | 3.77 |
|  | 3 | 24 | 0.15–0.48 | 0.31 | 0.10 | 0.16–225.41 | 33.21 | 75.87 | 0.05–31.48 | 4.30 | 7.00 |
|  | 0 | 24 | 0.13–0.71 | 0.23 | 0.13 | 0.10–224.00 | 26.36 | 64.68 | 0.27–44.99 | 6.07 | 11.37 |
| Kruskal Wallis test | | | Chi-square = 46.24, df = 2, p < 0.0001 | | | Chi-square = 24.70, df = 2, p < 0.0001 | | | Chi-square = 2.90, df = 2, p = 0.23 | | |

BEN = Benin, CMR = Cameroon, SEN = Senegal, P = parboiled, W = white, NT = non-treated, T = treated, min = minimum, max = maximum, std. Dev. = standard deviation. Obs. = observation.
